# Mathematical Modeling of COVID-19 Cases and Deaths and the Impact of Vaccinations during Three Years of the Pandemic in Peru

**DOI:** 10.3390/vaccines11111648

**Published:** 2023-10-27

**Authors:** Olegario Marín-Machuca, Ruy D. Chacón, Natalia Alvarez-Lovera, Pedro Pesantes-Grados, Luis Pérez-Timaná, Obert Marín-Sánchez

**Affiliations:** 1 Departamento Académico de Ciencias Alimentarias, Facultad de Oceanografía, Pesquería, Ciencias Alimentarias y Acuicultura, Universidad Nacional Federico Villarreal, Calle Roma 350, Miraflores 15074, Peru; omarin@unfv.edu.pe; 2 Department of Pathology, School of Veterinary Medicine, University of São Paulo, Av. Prof. Orlando M. Paiva, 87, São Paulo 05508-270, Brazil; 3 Escuela Profesional de Genética y Biotecnología, Facultad de Ciencias Biológicas, Universidad Nacional Mayor de San Marcos, Av. Carlos Germán Amezaga 375, Lima 15081, Peru; natalia.alvarez@unmsm.edu.pe (N.A.-L.); luis.perez30@unmsm.edu.pe (L.P.-T.); 4 Unidad de Posgrado, Facultad de Ciencias Matemáticas, Universidad Nacional Mayor de San Marcos, Av. Carlos Germán Amezaga 375, Lima 15081, Peru; pedro.pesantes@unmsm.edu.pe; 5 Departamento Académico de Microbiología Médica, Facultad de Medicina, Universidad Nacional Mayor de San Marcos, Av. Carlos Germán Amezaga 375, Lima 15081, Peru

**Keywords:** infections, deaths, vaccines, correlation, logistic model, pandemic, SARS-CoV-2

## Abstract

The COVID-19 pandemic has caused widespread infections, deaths, and substantial economic losses. Vaccine development efforts have led to authorized candidates reducing hospitalizations and mortality, although variant emergence remains a concern. Peru faced a significant impact due to healthcare deficiencies. This study employed logistic regression to mathematically model COVID-19’s dynamics in Peru over three years and assessed the correlations between cases, deaths, and people vaccinated. We estimated the critical time (t_c_) for cases (627 days), deaths (389 days), and people vaccinated (268 days), which led to the maximum speed values on those days. Negative correlations were identified between people vaccinated and cases (−0.40) and between people vaccinated and deaths (−0.75), suggesting reciprocal relationships between those pairs of variables. In addition, Granger causality tests determined that the vaccinated population dynamics can be used to forecast the behavior of deaths (*p*-value < 0.05), evidencing the impact of vaccinations against COVID-19. Also, the coefficient of determination (R^2^) indicated a robust representation of the real data. Using the Peruvian context as an example case, the logistic model’s projections of cases, deaths, and vaccinations provide crucial insights into the pandemic, guiding public health tactics and reaffirming the essential role of vaccinations and resource distribution for an effective fight against COVID-19.

## 1. Introduction

The coronavirus disease 2019 (COVID-19) pandemic began in Wuhan, China, in December 2019 [1]. This disease is caused by severe acute respiratory syndrome coronavirus 2 (SARS-CoV-2). In the first week of the pandemic, asymptomatic cases barely reached 1%. Among infected people, 81% had mild symptoms, 14% were severe, and 5% were critical or fatal [2]. Thus, since its emergence, this virus has spread rapidly worldwide, causing more than 676 million infections, 6.8 million deaths, 13.3 billion total vaccine doses administered, and trillions of USD in economic losses [3,4].

This disease presents some particular characteristics that have favored its spread and catastrophic impact: (1) the etiological agent, an RNA virus, which is endowed with evolutionary mechanisms such as a high mutation rate and genetic recombination [5]; (2) its long incubation period, which allows asymptomatic infected people to transmit the virus [6]; (3) susceptible individuals with a higher risk of fatality, including the elderly, immunosuppressed, and people with underlying diseases [7]; (4) various sources of contagion, including droplets from sneezing, coughing, and contact with or ingestion of contaminated objects [8].

Since the first reports of increased community spread, developing vaccines against COVID-19 has been a global struggle, including the participation of both the public and private sectors. Consequently, there are currently 382 vaccine candidates, including 199 in preclinical development and 183 in the clinical phase [9]. The emergency nature of the pandemic has stimulated the development of several vaccine platforms, including classical vaccines (i.e., live attenuated virus and inactivated virus) and new-generation vaccines (e.g., protein subunits, viral vectors, DNA, RNA, virus-like particles) [10].

Vaccine candidates that have successfully passed preclinical and clinical evaluations and been authorized for emergency use have significantly reduced hospitalizations and mortality rates [11]. However, the differences in the efficacy of the vaccines and the great mutational capacity of SARS-CoV-2 have allowed for the emergence of variants of concern with the ability of immunological evasion, leading to new waves of infection [12].

Peru was one of the countries most affected by the pandemic, due to deficiencies in its hospital system, and exhibited the highest infection–fatality ratio (IFR) [13]. The number of confirmed infections is over 4.5 million, while the deaths are over 220,000. The vaccines used in Peru are CoronaVac, AstraZeneca, Pfizer, Jansen, and Moderna. Over 89.5 million vaccine doses have been administered, and over 28.6 million people are fully vaccinated [3].

Mathematical modeling is a powerful tool that is used to study the spread of COVID-19 in many countries. Mathematical models can predict how the disease will spread over time by tracking the flow of individuals between different compartments, such as susceptible, infectious, recovered, and dead. Each of these types of models has its strengths and weaknesses [14]. SIR (susceptible–infected–recovered) models are relatively simple to understand and can be used to make quick predictions. However, they must consider the full complexity of the disease’s spread [15]. SEIR (susceptible–exposed–infected–recovered) models are more complex but can provide a more accurate picture of how a disease spreads [16,17]. SEIRD (susceptible–exposed–infected–recovered–dead) models are extensions of SEIR models that add a compartment for individuals who have died from the disease. This allows SEIRD models to track the entire course of an epidemic, from the early stages to the end [18,19].

Additionally, logistic models are curve-fitting models that can be used to fit a sigmoidal (S-shaped) curve to data. This curve is often seen in the spread of infectious diseases, as the number of cases typically increases slowly at first, then faster, and then slows down again as the population reaches herd immunity. One advantage of logistic models is that they are relatively simple to understand and use, and they can also be employed to predict an epidemic’s course. However, logistic models do not consider all of the factors that can influence the spread of a disease, such as the contact rate between individuals and the effectiveness of public health interventions [20,21]. Studies applying logistic regression have been successfully adapted to a wide range of COVID-19 research applications, including the assessment of clinical severity [22], risk analysis [23], the use of vaccines [24], and immune protection by neutralizing antibody levels [25], among others, highlighting the versatility and importance of this type of model.

The objective of this study was to use logistic regression to mathematically model the dynamics of COVID-19 in Peru during 30 months of the pandemic, encompassing cases, deaths, and people vaccinated. Furthermore, potential correlations were assessed between cases or deaths and the number of people vaccinated.

## 2. Materials and Methods

### 2.1. Data Collection

Raw data on infections, deaths, and people vaccinated in Peru were retrieved from the World Health Organization’s COVID-19 Dashboard [3]. These data included the period from 6 March 2020 to 20 March 2023. Data variables included total cases, total deaths, and people vaccinated.

To describe the epidemiological panorama of COVID-19 in Peru, we plotted the progression of the variables of cases, deaths, and people vaccinated throughout the selected period. The definition of these variables was as follows:

- Cases: Total confirmed cases of COVID-19. Counts can include probable cases, where reported (from 6 March 2020 to 20 March 2023).
- Deaths: Total deaths attributed to COVID-19. Counts can include probable deaths, where reported (from 6 March 2020 to 20 March 2023).
- People vaccinated: Total number of people who received at least one vaccine dose (from 9 February 2021 to 20 March 2023).

All data is available in the supplementary file (Table S1).

### 2.2. Mathematical Modeling

Mathematical modeling was based on the empirical modeling theory of Bronshtein and Semendiaev and applied to logistic regression for estimating the dynamics of COVID-19 cases in Peru, encompassing cases, deaths, and people vaccinated. The logistic model was derived from the Verhulst–Pearl logistic model, which describes growth that is initially exponential but slows down as the population nears its carrying capacity [26,27].

The formula to calculate the dynamics of COVID-19 in Peru describes a logistic dispersion of the following form:

(1) $$N=\frac{M}{\left(1+Q\times e^{-k\times t}\right)}$$

where “M” is the maximum number of cases, deaths, and people vaccinated, “Q” is a pre-exponential amount, “k” is a proportionality constant, “t” is the elapsed time (in days), and “N” is the number of cases, deaths, and people vaccinated, depending on the case.

The formula for calculating “M” for the three events involves three independent random values and their corresponding dependent values from the database, using the following formula:

(2) $$M=\frac{A\times B-I^{2}}{A+B-2I}$$

The first value (*A*) is the dependent variable value corresponding to the independent variable ($t_{1}$), where the behavior presents an inflection point. If the inflection (mean) value obtained is not an integer, the next integer value is taken (through rounding). If this latter value is not present in the data table, the next higher value displayed—more significant than the obtained value—is considered, including the linked value.

The second value (*B*) is the dependent variable value corresponding to the last value of the independent variable ($t_{2}$). The third value (I) is the dependent variable value related to the semi-sum of the independent variables $t_{1}$ and $t_{2}$, denoted by $t_{3}=\frac{t_{1}+t_{2}}{2}$. If the mean value obtained is not an integer, the next integer value is taken (through rounding), and the latter value is considered, including the linked value.

The determined value of M is then replaced in the logistic model. If the value obtained is not an integer, the next integer value is taken (through rounding). The logistic model is mathematically linearized, and the least squares method is applied to adopt the form $ln\left(\frac{M}{N}-1\right)\;lnQ+k\times t$; a linear equation y=A+Cx, where $y=ln\left(\frac{M}{N}-1\right)$, x=t, and A=lnQ.

The statistical process of linear regression can be performed by entering the ordered pairs (x,y) in the format $\left[\left(\frac{M}{N}-1\right)\right]$. Once all ordered pairs have been entered, we search for *lnQ* and *k*. The value of k is the slope of the linear equation; that is, the “C” value in the linear equation y=A+Cx, where the value of A is lnB and, therefore, $Q=e^{A}$. During the same linear regression process, we evaluated the correlation statistic Pearson’s “r”. To estimate the rates of cases, deaths, and people vaccinated (people/day) due to COVID-19 in Peru, we derived the determined logistic model, which takes the following form:

(3) $$\frac{dN}{dt}=\left[\frac{M\times Q\times k\times e^{-k\times t}}{{\left(1+Q\times e^{-k\times t}\right)}^{2}}\right]$$

To determine the critical time ($t_{c}$) at which the number of cases, deaths, and people vaccinated with respect to COVID-19 will be the maximum value, we derive Expression (3), set it equal to zero, and determine the word:

(4) $$t_{c}=-\frac{1}{k}\times \ln\left(\frac{1}{Q}\right)$$

If the value obtained is not an integer, the next integer value is taken (through rounding), and the latter value is considered for estimating the searched value in the original model.

We used the tidyverse package in the R programming language in Rstudio IDE [28]. Tidyverse is a set of R packages designed to import, transform, visualize, and model the information used in data science processes. It contains the ggplot2 package used to make the graphs in this article.

### 2.3. Statistical Analysis

We used the nortest and stats packages in the R programming language within R Studio IDE. Nortest is a collection of R functions specifically designed for conducting normality tests, while stats encompass R functions dedicated to statistical tests and data analysis. Correlation tests were conducted to evaluate whether vaccines reduce infections and deaths. Before performing these tests, a normality test was conducted for each population (cases, deaths, and people vaccinated).

Additionally, the tseries and lmtest packages were used because they provide a series of specific functions for time-series analysis. The data collected were presented in the form of time series, because this is a process of continuous counting of the new daily values and cumulative values of our populations. The time series for each of our populations were subjected to stationarity analysis and, subsequently, the causal relationship between the dynamics of infected and deceased with respect to the vaccinated was analyzed.

#### 2.3.1. Normality Tests for the Variables: Cases, Deaths, and People Vaccinated

To assess whether the data for each variable (cases, deaths, and people vaccinated) followed a normal distribution, hypothesis testing was conducted. These tests provide a *p*-value representing the probability of observing a data distribution similar to or even further from normality, assuming the null hypothesis indicates that the variable follows a perfect normal distribution in the population. If the *p*-value exceeds the significance level, there is insufficient evidence to reject the null hypothesis, suggesting that the variable follows a normal distribution [29]. Since the data in each population exceeded N>50, the Kolmogorov–Smirnov test was performed using the lillie.test function from the nortest package for each population.

Hypothesis test:

**Hypothesis 0** **(H0).** *The data follow a normal distribution.*

**Hypothesis 1** **(H1).** *The data do not follow a normal distribution.*

A significance level (*α*) of 0.05 was set.

#### 2.3.2. Correlation Test between People Vaccinated and Cases

Based on the normality test results, a correlation test was conducted using Spearman’s method, since the data in all populations did not follow a normal distribution [30]. The cor.test (vaccinations, daily_cases, method = “spearman”) function was employed to conduct the test.

Additionally, the cor (vaccinations, daily_cases, method = “spearman”) function was used to visualize the type of correlation (positive or negative), where a negative rho value indicates a negative correlation, while a positive rho value indicates a positive correlation.

Hypothesis test:

**Hypothesis 0** **(H0).** *The variables are not correlated.*

**Hypothesis 1** **(H1).** *The variables are correlated.*

A significance level (*α*) of 0.05 was set.

Correlation tests were conducted with real data and modeled data. For modeled data, the values for cases_velocity were employed instead of those for daily_cases.

#### 2.3.3. Correlation Test between People Vaccinated and Deaths

Based on the normality test results, a correlation test was conducted using Spearman’s method, since the data in all populations did not follow a normal distribution [30]. The cor.test (people vaccinated, daily_deaths, method = “spearman”) was used to conduct the test. Additionally, the cor (people vaccinated, daily_deaths, method = “spearman”) function was employed to visualize the type of correlation (positive or negative), where a negative rho value indicates a negative correlation, while a positive rho value indicates a positive correlation.

Hypothesis test:

**Hypothesis 0** **(H0).** *The variables are not correlated.*

**Hypothesis 1** **(H1).** *The variables are correlated.*

A significance level (*α*) of 0.05 was set.

Correlation tests were conducted with real data and modeled data. For modeled data, the values for deaths_velocity were employed instead of those for daily_deaths.

#### 2.3.4. Causality Tests for the Variables: Cases, Deaths, and People Vaccinated

In order to establish a causal relationship between the study variables (cases, deaths, and vaccinated), the Granger causality test was performed. This is a hypothesis test used to determine whether a time series is statistically and significantly useful for predicting the behavior of another time series [31,32,33].

This causality relationship, also known as Granger-causality, is because a cause precedes an effect, and because knowing how the history of one time series affects the dynamics of the other improves its prediction, i.e., strictly speaking, this causality relationship refers to the precedence and predictive capacity of one series over another. Therefore, when we state that a time series X causes another time series Y “in the Granger sense”, we mean that X is a statistically significant predictor of Y [31,32].

This test gives us a *p*-value from the F-statistic; if its value exceeds the given significance level, we can reject the null hypothesis, which suggests that time series X is useful for the prediction of series Y. The hypotheses and decision rule used are as follows:

**Hypothesis 0** **(H0).** *Time series X does not cause (in the Granger sense) time series Y.*

**Hypothesis 1** **(H1).** *Time series X is a causal predictor of time series Y.*

A significance level (*α*) of 0.05 was used.

#### 2.3.5. Stationarity Tests for the Variables: Cases, Deaths, and People Vaccinated

Granger causality testing requires the time series to be stationary. To verify this requirement, we performed the augmented Dickey–Fuller (ADF) test, which checks for stationarity between a pair of time series. This test analyzed the stationarity in each of the time series studied (cases, deaths, and people vaccinated). In this test, if the *p*-value is less than the significance level, then we reject the null hypothesis and suggest that the series is stationary. The hypotheses to test for a time series X are the following:

**Hypothesis 0** **(H0).** *Time series X is not stationary.*

**Hypothesis 1** **(H1).** *Time series X is stationary.*

A significance level (α) of 0.05 was used.

#### 2.3.6. Information Criteria for Determining the Lag-Orders

In the Granger test, the lag selected refers to the time lag that will be used to predict one time series from the other. On the other hand, the lag in the augmented Dickey–Fuller test refers to the number of lags in the difference of the time series, which is used to assess its stationarity. Therefore, the optimal lag selected for the Granger test cannot be used directly in the augmented Dickey–Fuller test.

Both the Granger causality test and the augmented Dickey–Fuller test require that a lag value be specified. For this purpose, the Akaike information criterion (AIC) and the Bayesian information criterion (BIC) were used. These criteria are used to compare different models with different lag values and determine which one provides the best fit to the data, because their performance depends on the sample size and the lag order [32,34]. The information criteria penalize models that are more complex in terms of the number of lags included [35]. Therefore, by calculating these criteria for various lag values, one can identify the model that has the lowest value of AIC or BIC, which suggests that it is the best-fitting model for one’s data, i.e., the lag value that minimizes AIC or BIC is the one that is considered optimal according to these criteria.

#### 2.3.7. Comparison of Modeled Variables against Real Data

To verify whether the mathematical model explained the behavior of the real data, the coefficient of determination $R^{2}$ was calculated [30]. $R^{2}$ represents the proportion of the total variability in the response variable explained by the model. A value of $R^{2}$ closer to 1 indicates a good fit of the model, suggesting that the model explains a large portion of the response variable’s variability. On the other hand, an $R^{2}$ value close to 0 indicates that the model does not adequately explain the variability of the response variable. The following mathematical formula was employed to calculate $R^{2}$: $R^{2}=1-\left(SSR/SST\right)$, where SSR (sum of squares residual) is the sum of the squared differences between the predicted values from the model and the actual values of the response variable, while SST (sum of squares total) is the sum of the squared differences between the actual values of the response variable and its mean.

## 3. Results

### 3.1. Epidemiological Panorama of COVID-19 in Peru

**Figure 1:**
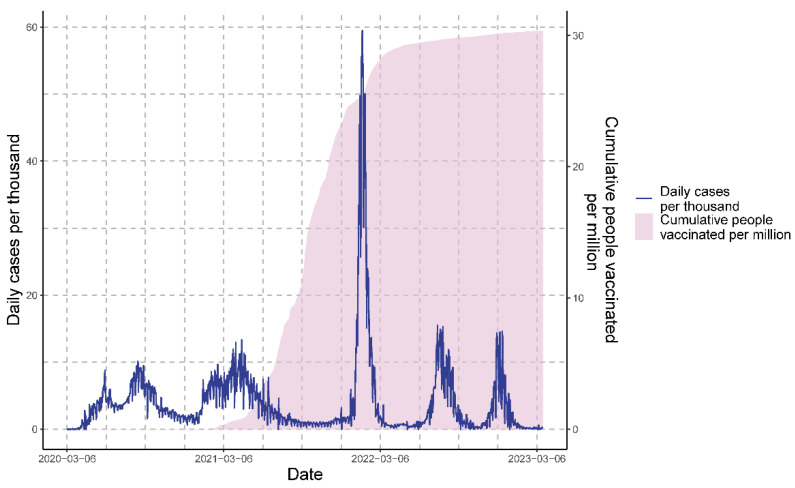
Epidemiological overview of daily cases and cumulative people vaccinated in three years of the COVID-19 pandemic in Peru.

The first case of COVID-19 in Peru was confirmed on 6 March 2020. Since then, the number of cases has been increasing. Five epidemiological waves reaching or exceeding ten cases (infections) per thousand were observed, with peaks around September 2020, April 2021, February and July 2022, and January 2023 (Figure 1).

**Figure 2:**
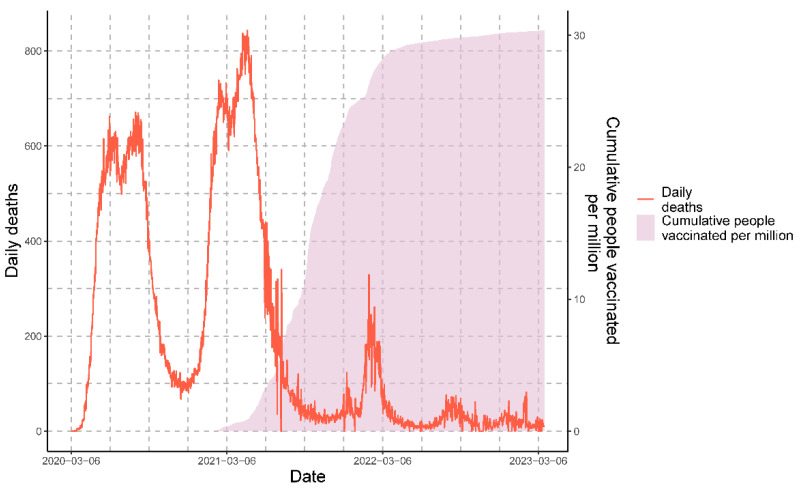
Epidemiological overview of daily deaths and cumulative people vaccinated in three years of the COVID-19 pandemic in Peru.

Regarding deaths, the temporal progression of COVID-19 deaths in Peru from March 2020 to March 2023 is shown in Figure 2. The graph depicts the three distinct waves of death. Each wave is characterized by reaching or surpassing a threshold of 200 daily deaths.

**Figure 3:**
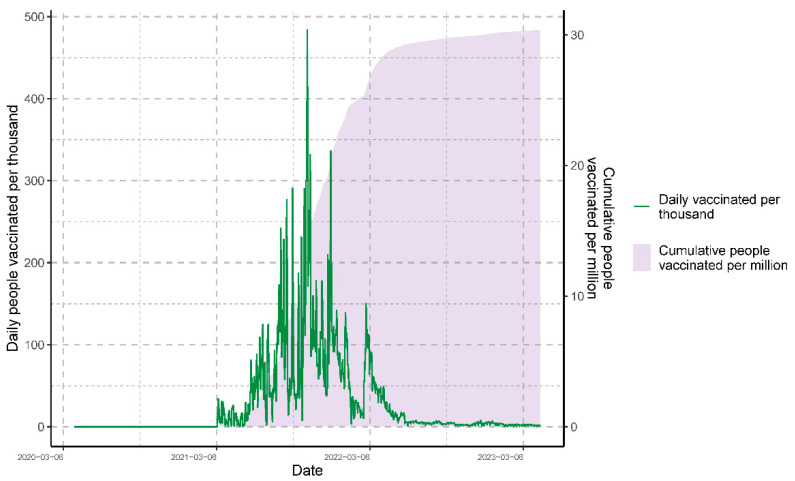
Epidemiological overview of daily and cumulative people vaccinated in three years of the COVID-19 pandemic in Peru.

The vaccination process in Peru began on 9 February 2021 and has taken into account the synchronic data from the beginning of the COVID-19 pandemic until 20 March 2023. The data of new daily and accumulated vaccinations are shown in Figure 3. This graph shows different vaccination cycles, which are characterized by peaks exceeding 100,000 daily vaccinations.

**Figure 4:**
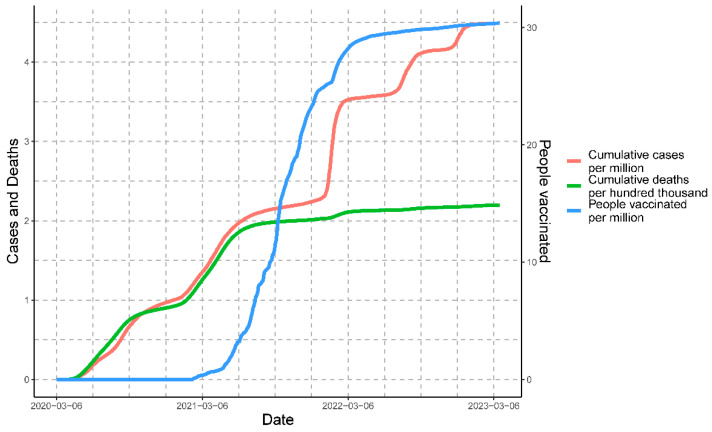
Epidemiological overview of cumulative cases, deaths, and people vaccinated in three years of the COVID-19 pandemic in Peru.

The epidemiological scenario for the cumulative data for the three variables simultaneously can be observed in Figure 4. The graph shows the temporal progression of COVID-19 cases, deaths, and people vaccinated in Peru from March 2020 to March 2023. The graph depicts the cumulative cases and people vaccinated per million, along with the cumulative deaths per hundred thousand.

### 3.2. Mathematical Modeling

The obtained parameters were summarized using the above methodology and procedures (Table 1). The table shows the values for cases, deaths, and people vaccinated obtained by the logistic model.

The detailed procedures for applying the model for cases, deaths, and people vaccinated are delineated in the following subsections:

#### 3.2.1. Mathematical Modeling for Cases

The first value: $t_{1}=642$ days; corresponds to A=2,245,146 people.

The second value, $t_{2}=1109$ days; corresponds to B=4,489,377 people.

The third value: $t_{3}=\frac{642\;+\;1109}{2}=872$ days; corresponds to I=3,889,092 people.

Now, replacing in (2): $M=\frac{2,245,146\;\times \;4,489,377\;-\;3,889,029^{2}}{2,245,146\;+\;4,489,377\;-\;2\;\times \;3,889,029}=4,834,759$ people.

Then, the model $Q=\frac{M}{\left(1\;+\;B\;\times \;e^{-k\times t}\right)}$ can be written as $Q=\frac{4,874,759}{\left(1\;+\;B\;\times \;e^{-k\times t}\right)}$.

Applying the least squares method to the expression $ln\left(\frac{4,834,759}{Q}-1\right)=A+k\times t$, we can obtain the prediction or estimation model.

(5) $$Q=\frac{4,834,759}{\left(1+66.6730\times e^{-0.0067\times t}\right)}$$

With a correlation coefficient r=−0.8554, and deriving Equation (5), we can obtain the equation for the speed of infected people, expressed by Equation (6):

(6) $$\frac{dQ}{dt}=\left[\frac{2,159,730.84\times e^{-0.0067\times t}}{{\left(1+66.6730\times e^{-0.0067\times t}\right)}^{2}}\right]$$

Deriving Equation (5) and equaling to zero, we can determine the critical time (tc) for which the velocity of the infected people is maximum.

$$t_{c}=-1/0.0067ln\left(\frac{1}{66.6730}\right)=627\;\mathrm{days}$$

**Figure 5:**
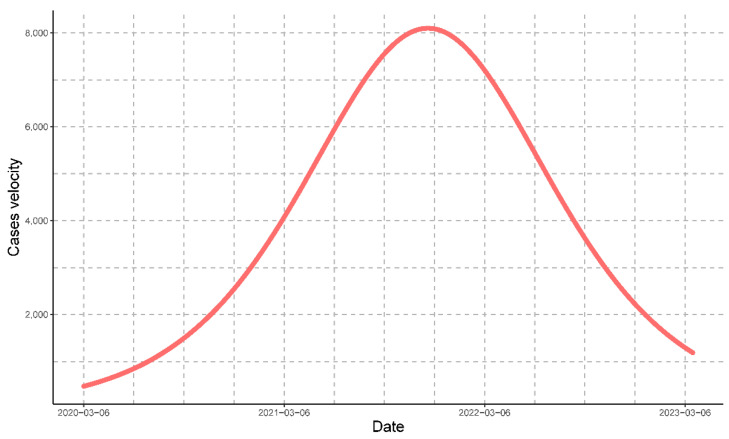
The velocity of the progression of COVID-19 cases represented by the red line. The graph depicts the critical time of the progression at which Peru had the maximum daily value for cases.

Then, $t_{c}=627$ days, and the maximum speed is ${\left(\frac{dN}{dt}\right)}_{máx}=8098$ people/day (Figure 5).

For COVID-19 in Peru, the maximum rate of estimated cases was on 23 November 2021; the number of estimated cases was determined by Equation (5), and the rate of change or rate of estimated cases was determined by Equation (6).

#### 3.2.2. Mathematical Modeling for Deaths

The first value: $t_{1}=343$ days; corresponds to A=110,184 people.

The second value: $t_{2}=1109$ days; corresponds to A=219,648 people.

The third value: $t_{3}=\frac{343\;+\;1109}{2}=726$ days; corresponds to I=210,672 people.

Now, replacing in (2): $M=\frac{110,184\;\times \;219,684\;-\;210,672^{2}}{110,184\;+\;219,684\;-\;2\;\times \;210,672}=220,528$ people.

Then, the model: $Q=\frac{220,528}{\left(1\;+\;B\;\times \;e^{-k\times t}\right)}$.

Applying the least squares method to the expression

$ln\left(\frac{220,528}{Q}-1\right)=A+k\times t$, we can obtain the prediction or estimation model:

(7) $$Q=\frac{220,528}{\left(1+25.1760\times e^{-0.0083\times t}\right)}$$

with a correlation coefficient r=−0.8902. Deriving Equation (7), we can obtain the equation for the speed of dead people, expressed by Equation (8):

(8) $$\frac{dQ}{dt}=\left[\frac{46,081.7073\times e^{-0.0083\times t}}{{\left(1+25.1760\times e^{-0.0083\times t}\right)}^{2}}\right]$$

Deriving Equation (7) and equaling to zero, we can determine the critical time (tc) for which the velocity of the dead people is maximum.

$$t_{c}=-1/0.0083ln\left(\frac{1}{25.1760}\right)\;=389\;\mathrm{days}$$

**Figure 6:**
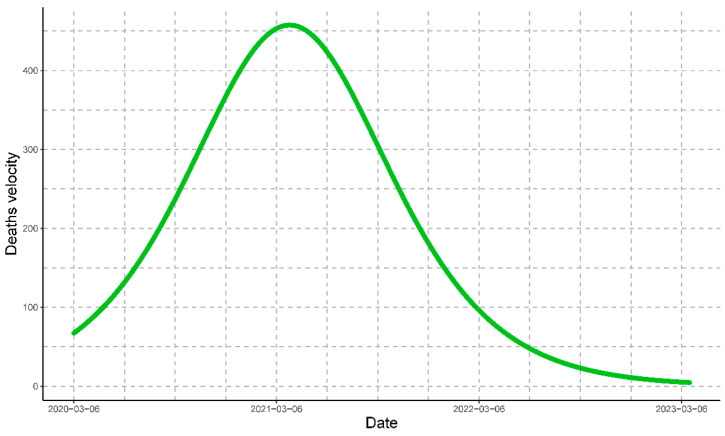
The velocity of the progression of COVID-19 deaths represented by the green line. The graph depicts the critical time of the progression at which Peru had the maximum daily value for deaths.

Then, $t_{c}=389$ days, and the maximum speed is ${\left(\frac{dN}{dt}\right)}_{máx\;}=459$ people/day (Figure 6).

For COVID-19 in Peru, the maximum rate of estimated fatalities was on 30 November 2021; the number of estimated deaths was determined by Equation (7), and the rate of change or rate of estimated deaths was determined by Equation (8).

#### 3.2.3. Mathematical Modeling for People Vaccinated

The first value: $t_{1}=222$ days; corresponds to A=15,348,800 people.

The second value: $t_{2}=769$ days; corresponds to B=30,774,977 people.

The third value: $t_{3}=\frac{222\;+\;769}{2}=496$ days; corresponds to I=29,556,095 people.

Now, replacing in (2): $M=\frac{15,348,800\;\times \;30,774,977\;-\;29,556,095^{2}}{15,348,800\;+\;30,774,977\;-\;2\;\times \;29,556,095}=30,429,043$ people. Then, the model $Q=\frac{M}{\left(1\;+\;B\;\times \;e^{-k\times t}\right)}$ can be written as $Q=\frac{30,429,043}{\left(1\;+\;B\;\times \;e^{-k\times t}\right)}$.

Applying the least squares method to the expression

$ln\;\left(\frac{30,429,043}{Q}-1\right)=A+k\times t$, we can obtain the prediction or estimation model:

(9) $$Q=\frac{30,429,043}{\left(1+35.0510\times e^{-0.0133\times t}\right)}$$

with a correlation coefficient r=−0.722. Deriving Equation (9), we can obtain the equation for the speed of people vaccinated, expressed by Equation (10):

(10) $$\frac{dQ}{dt}=\left[\frac{14,185,359.54\times e^{-0.0133\times t}}{{\left(1+35.0510\times e^{-0.0133\times t}\right)}^{2}}\right]$$

Deriving Equation (9) and equaling to zero, we can determine the critical time ($t_{c}$) for which the velocity of the people vaccinated is maximum:

$$t_{c}=-1/0.0133ln\left(\frac{1}{35.0510}\right)=268\;\mathrm{days}$$

**Figure 7:**
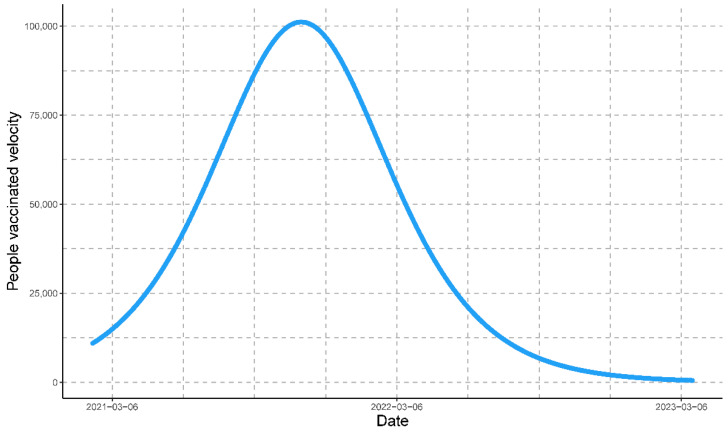
The velocity of the progression of people vaccinated for COVID-19 represented by the blue line. The graph depicts the critical time of the progression at which Peru had the maximum daily value for people vaccinated.

Then, $t_{c}=268$ days, and the maximum speed is ${\left(\frac{dN}{dt}\right)}_{máx}=101,175$ people/day (Figure 7).

**Figure 8:**
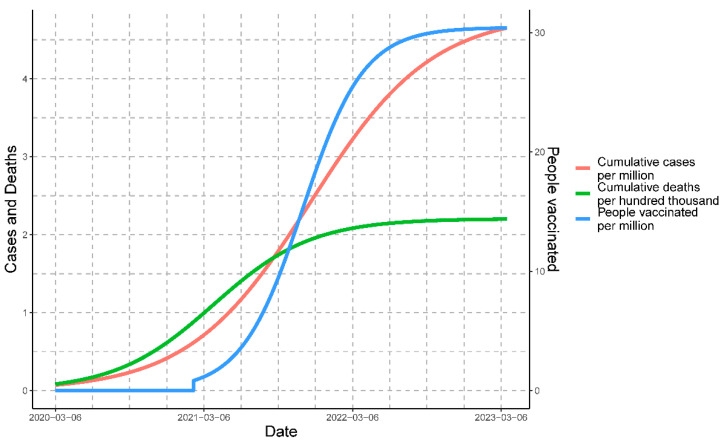
Epidemiological temporal progression of COVID-19 cases, deaths, and people vaccinated in Peru from March 2020 to March 2023 using a logistic method. The graph depicts the cumulative cases and people vaccinated per million, along with the cumulative deaths per hundred thousand.

The maximum estimated rate of people vaccinated against COVID-19 in Peru was on 4 November 2021; the estimated number of people vaccinated was determined by Equation (9), and the estimated rate of change or rate of people vaccinated was determined by Equation (10). The epidemiological scenario based on the modeled data for the three variables simultaneously can be observed in Figure 8.

**Figure 9:**
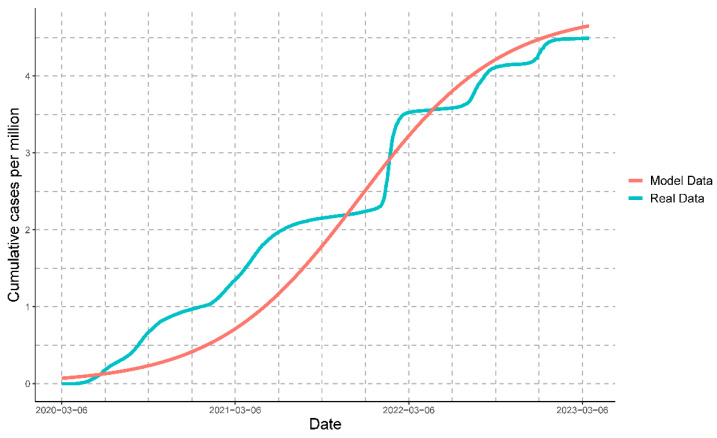
Comparison of the real data of cases with the modeled data of cases. Every million cases represents cumulative cases.

**Figure 10:**
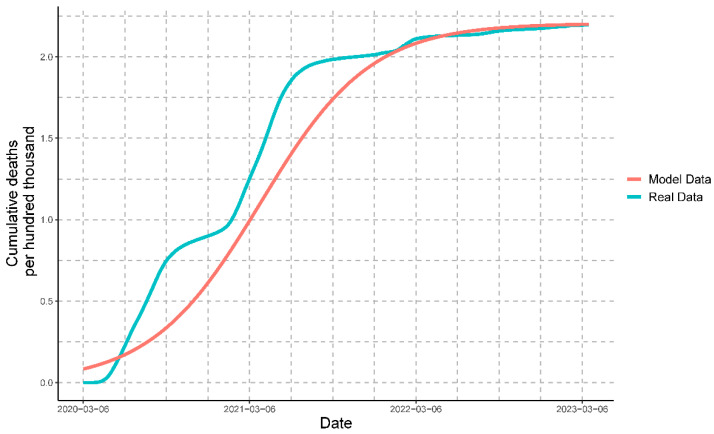
Comparison of the real data of deaths with the modeled data of deaths. Every hundred thousand deaths represents cumulative deaths.

**Figure 11:**
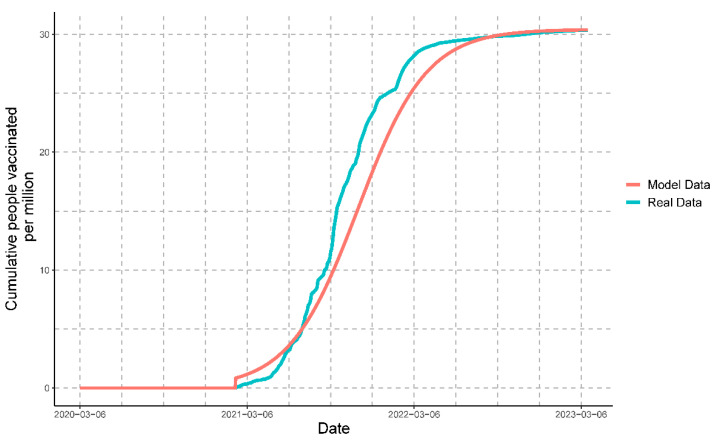
Comparison of the real data of people vaccinated with the modeled data. Every million people vaccinated represents cumulative vaccinations.

Once we had mathematically modeled all of the variables, it was possible to compare the progression of the real data with that of the modeled data for cases (Figure 9), deaths (Figure 10), and people vaccinated (Figure 11).

### 3.3. Statistical Analysis

#### 3.3.1. Normality Tests for the Variables: Cases, Deaths, and People Vaccinated

The data for all three populations (cases, deaths, and people vaccinated) did not exhibit a normal distribution. The *p*-value for the “people vaccinated” data was $<2.2\times 10^{-16}$, indicating rejection of the null hypothesis and that the data did not follow a normal distribution. The *p*-value for the “cases” data was <2.2 × 10^−16^, indicating rejection of the null hypothesis and that the data did not follow a normal distribution. The *p*-value for the “deaths” data was $<2.2\times 10^{-16}$, indicating rejection of the null hypothesis and that the data did not follow a normal distribution.

#### 3.3.2. Correlation Test between People Vaccinated and Cases

The correlation test was conducted to evaluate whether the number of people vaccinated influenced the decrease in the number of infections. For real data, the *p*-value obtained was $<2.2\times 10^{-16}$, rejecting the null hypothesis and indicating a correlation between the variables. The cor() function yielded a rho value of −0.4001837, indicating a negative correlation. This negative correlation implies that an increased number of people vaccinated corresponds to a decrease in cases. For modeled data, the results were similar; the *p*-value obtained was $<2.2\times 10^{-16}$, rejecting the null hypothesis and indicating a correlation between the variables. The cor() function yielded a rho value of −0.4204788, indicating a negative correlation. This negative correlation implies that an increased number of people vaccinated corresponds to a decrease in cases.

#### 3.3.3. Correlation Test between People Vaccinated and Deaths

The correlation test was conducted to evaluate whether the number of people vaccinated influenced the decrease in deaths. For real data, the *p*-value was <2.2 × 10^−16^, rejecting the null hypothesis and indicating a correlation between the variables. The cor() function yielded a rho value of −0.7530406, indicating a negative correlation. This negative correlation implies that an increased number of people vaccinated leads to decreased deaths. For modeled data, the *p*-value was <2.2 × 10^−16^, rejecting the null hypothesis and indicating a correlation between the variables. The cor() function yielded a rho value of −0.9977608, indicating a negative correlation. This negative correlation implies that an increased number of people vaccinated leads to decreased deaths.

Furthermore, it should be noted that the rho value obtained in the cor() test conducted for the people vaccinated and deaths populations was lower than the rho value obtained in the cor() test conducted for the people vaccinated and cases, indicating that the negative correlation for people vaccinated and deaths was stronger than the negative correlation for people vaccinated and cases. This result was obtained in a similar way for both the real data and the modeled data. Therefore, increasing the number of people vaccinated has a greater impact on reducing deaths than reducing cases.

#### 3.3.4. Information Criteria for Determining the Lag Orders

In determining the lag order for the augmented Dickey–Fuller stationarity test, each of the time series (cases, deaths, and people vaccinated daily) was subjected to a different lag order (from 1 to 25), and the values of AIC and BIC corresponding to each lag were obtained, choosing the lowest of them. These results are shown in Table 2.

The following lag-order values were selected for the time series of vaccinated, cases, and deceased: 9, 10, and 16, respectively.

**Figure 12:**
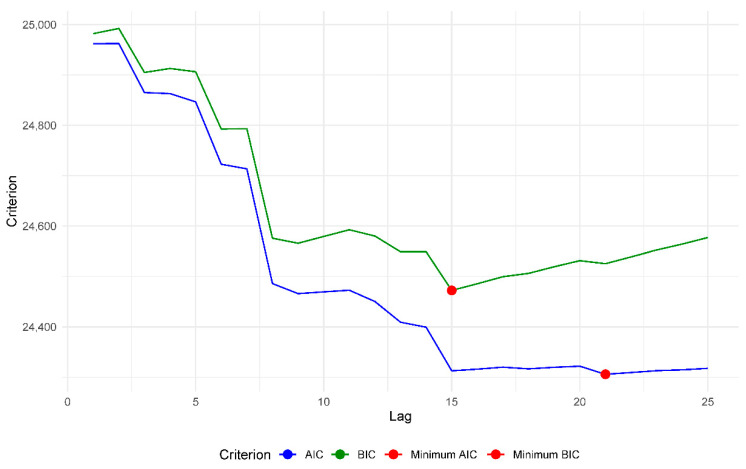
Plot between the different lags evaluated by the AIC and BIC criteria for the pair of time series deaths—people vaccinated.

In the case of the lag order for the Granger causality test, the minimum values of the AIC and BIC criteria found are shown in Figure 12 and Table 3, and all of the values found for the lag order range from 1 to 25 for the deaths–people vaccinated series.

In this case, we selected a lag order of 15 for both pairs of series.

#### 3.3.5. Stationarity Tests for the Variables: Cases, Deaths, and People Vaccinated

The time series for all three populations (cases, deaths, and people vaccinated) can be considered stationary. According to the Dickey–Fuller test augmented with lag-order values of 9 (vaccinated), 10 (cases), and 16 (deaths), using the adf.test() function of the tseries package, we found *p*-values < 0.01 (that is, *p*-values < 0.05 (significance level)) for all three populations; therefore, we can reject the null hypothesis and establish that the three time series are stationary, i.e., they exhibit a constant variance over time.

#### 3.3.6. Causality Tests between the Variables: Cases, Deaths, and People Vaccinated

Since the three time series were stationary, we proceeded to perform the Granger causality test for two pairs of series: cases–vaccinated and deaths–vaccinated. We analyzed both causality directions in each pair of series with a lag order of 15, using the grangertest (X, Y, order) function of the lmtest package; the following table (Table 4) shows the results:

Due to the *p*-value in the causality direction vaccinated → deaths being less than the significance level (*α* = 0.05), we can reject the null hypothesis, i.e., the values of the vaccinated people can be used to predict the values of the deaths. The other causality direction showed a *p*-value > 0.05; in these cases, we cannot reject the null hypothesis, i.e., there is no Granger causality between them.

#### 3.3.7. Comparison of Modeled Variables against Real Data

The comparison of the real data with the data obtained by the mathematical model for cases, deaths, and people vaccinated resulted in the following coefficients of determination ($R^{2}$): 0.9263022, 0.9099109, and 0.9814848, respectively. These values indicate that the mathematical model strongly represents the real data for all of the variables.

## 4. Discussion

The COVID-19 pandemic was an unprecedented catastrophe due to the emergence of the first pandemic-causing coronavirus and its uncontrollable spread, significantly mitigated only by using vaccines. Other contemporary social, political, and economic factors also influenced the impact. The intricate interplay of biological and non-biological factors contributed significantly to the transmission and dissemination of SARS-CoV-2, causing millions of cases and deaths worldwide. Due to the underlying dynamics of COVID-19, which exhibit variation across different geographical regions, we endeavored to employ mathematical modeling, specifically logistic regression, to analyze the dynamics of COVID-19 in Peru during three years of the pandemic period, encompassing cases, deaths, and people vaccinated. Additionally, we assessed potential correlations between cases or deaths and the number of people vaccinated.

Predicting and understanding infectious diseases’ behavior in epidemiology is critical for effective public health interventions and policymaking. Among the various modeling approaches, the logistic mathematical model has proven valuable for its simplicity, adaptability, and ability to capture the essential dynamics of disease progression in a wide range of COVID-19 research applications [22,23,24,25].

Mathematical modeling, specifically logistic regression, helps elucidate the dynamics of the COVID-19 pandemic in Peru over three years, encompassing data on cases, deaths, and people vaccinated. This research is further justified by the unique challenges posed by COVID-19, such as its rapid global spread, high mutation rate, and the emergence of variants that can evade immunity. While vaccines have been a pivotal tool in mitigating the pandemic’s effects, disparities in their efficacy and the virus’s mutational capabilities necessitate comprehensive modeling to predict future trends and inform public health strategies. Additionally, many publications endorse the logistic model’s usage for predicting COVID-19 cases, deaths, and vaccinations [36,37,38,39,40].

This study’s approach, rooted in the empirical modeling theory, employed logistic regression to estimate the dynamics of COVID-19 cases, deaths, and people vaccinated in Peru. The choice of a logistic model derived from the Verhulst–Pearl logistic model is apt for capturing growth patterns that start exponentially but decelerate as they approach a carrying capacity [27]. Such models are particularly suited for infectious diseases’ spread, where initial growth is rapid but slows down as factors like herd immunity come into play [14]. The detailed formulae, including the logistic dispersion and the method to calculate the maximum numbers of cases, deaths, and people vaccinated, offer a comprehensive mathematical framework. However, as highlighted by Kumari and Sharma [41], it would be beneficial to provide a rationale for the choice of specific parameters and constants, ensuring that the model’s assumptions align with the real-world dynamics of the disease.

In the logistic model, the critical time (tc) represents the maximum rate at which the population experiences infections, deaths, or vaccinations. It also signifies the inflection point of the logistic curve. From this point onward, the growth rate of the three cumulative populations begins to decrease, eventually stabilizing at their respective maximum values (i.e., the maximum numbers of cases, deaths, and people vaccinated). These critical times hold immense significance because they mark a reduction in the rate of infection or mortality, indicating that the epidemic is starting to come under control. It is imperative to maintain the conditions that foster this trend, such as increasing primary care, promoting preventive measures against contagion, and so on. Conversely, in the case of vaccinated individuals, this critical point suggests a change in vaccination policy. The vaccination rate has slowed down, which could be attributed to factors like vaccine shortages, saturation of the eligible population, or other causes. In addition, the implications of vaccination usage in our study help us to better understand its impact on the progression of the pandemic. This can indirectly provide insights into vaccine efficacy, potential resistance, and the need for booster doses or new formulations. Such knowledge can influence vaccine development, administration strategies, and patient counseling. From a broader public health perspective, these findings can guide strategic planning, allocation of resources, and public communication efforts.

This study’s utilization of the R programming language, and specifically the tidyverse package, is commendable [42]. Tidyverse is a robust toolset for data science processes, and its inclusion ensures rigorous data handling and visualization. However, as Mishra et al. noted [43], the statistical analysis section could benefit from a more in-depth exploration of the data’s characteristics before applying specific tests. While the normality tests and hypothesis testing are well structured, providing some descriptive statistics or preliminary visualizations would be advantageous to give readers a clearer understanding of the data’s distribution.

The correlation tests, focusing on the relationship between people vaccinated and cases or deaths, are crucial for understanding the vaccine’s impact. Spearman’s method is an appropriate choice given the non-normal distribution of the data [30]. However, discussing the implications of the correlation results in the context of public health and vaccination strategies would be beneficial. The present study identified vaccinations’ impact as negative correlations between vaccinated people and cases or deaths. Furthermore, the effect of vaccinations on reducing deaths was more pronounced, exhibiting a stronger negative correlation. The correlation concerning cases can be explained by the fact that vaccines do not prevent infections but, rather, mitigate them through a gradual decrease in antibodies among vaccinated individuals and new variants of SARS-CoV-2 with immune-evasion characteristics [11,12]. However, it is important to note that correlation only shows a reciprocal relationship between two variables and does not imply causality.

To determine the causality effect between two variables, some statistical strategies have been developed; for example, propensity score matching (PSM) is widely used in the epidemiological context when it is desired to minimize the bias of non-randomized studies and to determine the effects of control measures or treatments on populations of interest; however, this methodology is essentially applicable only to cross-sectional studies and requires cofounders or covariables to perform a robust study [44,45,46,47,48]. In addition, some methodologies have been implemented in time-varying treatment or exposure, but this methodology could be inappropriate [49,50]. Considering the nature of the variables addressed in this study, we implemented the Granger test to evaluate causality. In general, if the relevant variables and the relationships between them are known and can be formalized by means of vector autoregressive models (i.e., models that consider the past and present relationships of the variables, generally represented as difference equations), then the Granger test can be useful to study the level of predictability of one variable from another variable [51]. Since our data are in the form of time series (i.e., discrete values at regular, finite intervals, and with stationary characteristics), the Granger test can be applied. The Granger causality test allowed us to determine with statistical significance that the vaccinated population dynamics can be used to forecast the behavior of deaths. However, vaccination does not seem to have a direct effect on contagion and the spread of the disease; this is consistent with the characteristics of the vaccines applied in Peru, which were designed with the purpose of reducing the severity and, therefore, mortality, but not the contagion of the disease.

Despite the above, we must bear in mind that the causality test used is not free of bias, and that even when all of the requirements for its application are met, the results of the causality test must be interpreted in conjunction with the other components (i.e., the variables and relationships between them) of the system, in addition to the fact that nonlinear relationships between variables tend to diminish its reliability. Moreover, it does not take into account the effect of a third time series that could actually cause the behavior observed when analyzing pairs of populations [31,33]. In the context of our study, although a predictive effect of the dynamics of the vaccinated population with respect to the number of deaths was determined, the level of that effect was not estimated in comparison with other health measures, such as the implementation of pharmacological treatment for symptomatic patients, isolation, social distancing, the use of mechanical protectors, genetic and behavioral factors, etc. On the other hand, we should mention that since the model based on differential equations is a limiting case of equations in differences, causality in the Granger sense would give us evidence and indirect support to explicitly state this relationship in a mathematical model in differential equations with two or more variables and, therefore, extend the logistic model proposed to characterize the dynamics of each population.

Comparing modeled variables against real data using the coefficient of determination, R^2^, is critical in validating the model’s accuracy [52]. An R^2^ value close to 1 would indeed suggest a good fit, but as Härdle and Simar [53] emphasized, it is essential to interpret this in the context of the disease’s dynamics. Even a model with a high R^2^ might have limitations, especially if it does not account for external factors like public health interventions or behavioral changes in the population. It would be beneficial to juxtapose the R^2^ value with other goodness-of-fit metrics and perhaps compare the logistic model’s performance with other potential modeling approaches.

The inherent nature of the logistic model, which captures the sigmoidal (S-shaped) curve often seen in infectious diseases’ spread, makes it an appropriate choice. The initial slow rise, followed by a rapid increase and eventual plateauing, mirrors the real-world progression of many epidemics, including COVID-19 [54]. This is particularly significant given the unpredictable nature of the virus’s spread, influenced by factors such as public health interventions, behavioral changes, and vaccination rates. The logistic model’s ability to predict the potential carrying capacity (i.e., the maximum number of cases, deaths, or people vaccinated) is useful for policymakers and health officials to anticipate healthcare needs and strategize interventions.

Complex compartmental models like SIR, SEIR, and SEIRD offer detailed insights into disease dynamics, but they often require many parameters and assumptions. Conversely, the logistic model provides a straightforward approach to understanding the general trend of the epidemic without delving into the intricacies of disease transmission dynamics [14]. This makes it a powerful tool for quick assessments and predictions, especially when timely decisions are paramount.

While the logistic model offers many advantages, it is crucial to recognize its limitations. The model inherently assumes a symmetrical rise and fall, which might only sometimes align with real-world data, especially in the face of external interventions or the emergence of new variants [20]. Moreover, the model does not account for factors like contact rates between individuals or the effectiveness of public health measures. Therefore, while the logistic model provides a valuable overview, it should ideally be used with other models or data sources to better understand the epidemic’s dynamics.

Peru’s unique challenges during the pandemic have made it a focal point for international professionals. The nation presented the highest infection–fatality ratio during the most catastrophic months of the pandemic worldwide. Coupled with the challenges introduced by its healthcare system’s limitations, including the lesser quantities of mechanical respirators and intensive care units in the region, Peru offers a compelling case study for managing an outbreak under strained circumstances. Insights gained from Peru can be invaluable for nations with similar healthcare infrastructure or those facing similar external challenges. Moreover, understanding the effects of the pandemic on diverse populations, such as the Amazonian indigenous community in Peru, can shed light on disease dynamics in varied sociocultural contexts [55]. These findings can guide tailored interventions, resource allocation, and policymaking in other regions, emphasizing the universal relevance of localized studies [56].

## 5. Conclusions

This study concludes that mathematical modeling, particularly logistic regression, provided a valuable tool for analyzing the dynamics of COVID-19 in Peru over three years, focusing on cases, deaths, and vaccinations.

The identified critical times represented the maximum rates of cases, deaths, and vaccinations, signifying a shift in the epidemic’s dynamics towards stabilization. The significance of these points influences the understanding of disease dynamics by identifying the events that occurred before and after those rates, enabling timely measures for better pandemic control.

Negative correlations were observed between the number of people vaccinated and both cases and deaths, indicating a reciprocal relationship between these variables. Furthermore, according to the statistical evidence, it can be concluded that the dynamics of the vaccinated population is a good predictor to determine the behavior of the deceased; however, the effects of other variables (e.g., pharmacological treatment and non-pharmacological measures) as possible predictors of the deceased cannot be ruled out.

While the logistic model offered simplicity and quick assessments, it had inherent limitations when applied to real-world scenarios, such as assuming a symmetrical rise and omitting more complex or circumstantial variables.

Finally, insights from Peru’s unique pandemic challenges, including healthcare deficiencies and diverse populations, can provide valuable lessons for countries with similar infrastructure or external challenges. This can guide tailored interventions, resource allocation, and policymaking, emphasizing their universal relevance.

## Figures and Tables

**Table 1:** Basic parameters obtained by the logistic model.

Parameter	Cases	Deaths	People Vaccinated
*t*_1_	642	343	222
*A*	2,245,146	110,184	15,348,800
*t*_2_	1109	1109	769
*B*	4,489,377	219,648	30,374,977
*t*_3_	876	726	496
*I*	3,889,029	210,672	29,526,095
*M*	4,834,759	220,528	30,429,043
*a*	4.1998	3.2259	3.5568
*Q*	66.673	25.176	35.051
*k*	−0.0067	−0.0083	−0.0133
*t_c_*	627	389	268
Maximum speed at *t_c_*	8098	459	101,175
Maximum value at *t_c_*	2,418,709	141,432	19,890,913

**Table 2:** Criterion information results for the optimal lag order used in the augmented Dickey–Fuller test.

Time Series	Lag-Order AIC	AIC Value	Lag-Order BIC	BIC Value
People vaccinated	9	24,998.81	9	25,053.94
Cases	10	19,654.12	10	19,714.27
Deaths	16	10,476.19	15	10,562.1

**Table 3:** Criterion information results for the optimal lag order used in the Granger causality test.

Pair of Time Series	Lag-Order AIC	AIC Value	Lag-Order BIC	BIC Value
Deaths–people vaccinated	21	24,296.05	15	24,464.87
Cases–people vaccinated	21	24,305.82	15	24,472.33

**Table 4:** Summary results for the Granger causality test in each analyzed direction.

Pair of Time Series	Deaths → Vaccinated	Vaccinated → Deaths	Cases → Vaccinated	Vaccinated → Cases
*p*-Value	0.1828	0.01608	0.6495	0.9276

## Data Availability

Raw data on infections, deaths, and vaccinations for Peru were retrieved from the World Health Organization’s COVID-19 Dashboard [3]. These data included the period from 6 March 2020 to 20 March 2023.

## Supplementary Materials

The following supporting information can be downloaded at: https://www.mdpi.com/article/10.3390/vaccines11111648/s1, Table S1: Real and modeled data on infections, deaths, and people vaccinated in Peru.
